# Supplementary figures and images for: A lightweight and secure online/offline cross-domain authentication scheme for VANET systems in Industrial IoT
Source: PeerJ Comput Sci. 2021 Dec 10;7:e714. doi: 10.7717/peerj-cs.714 (PMC8670398; doi:10.7717/peerj-cs.714)

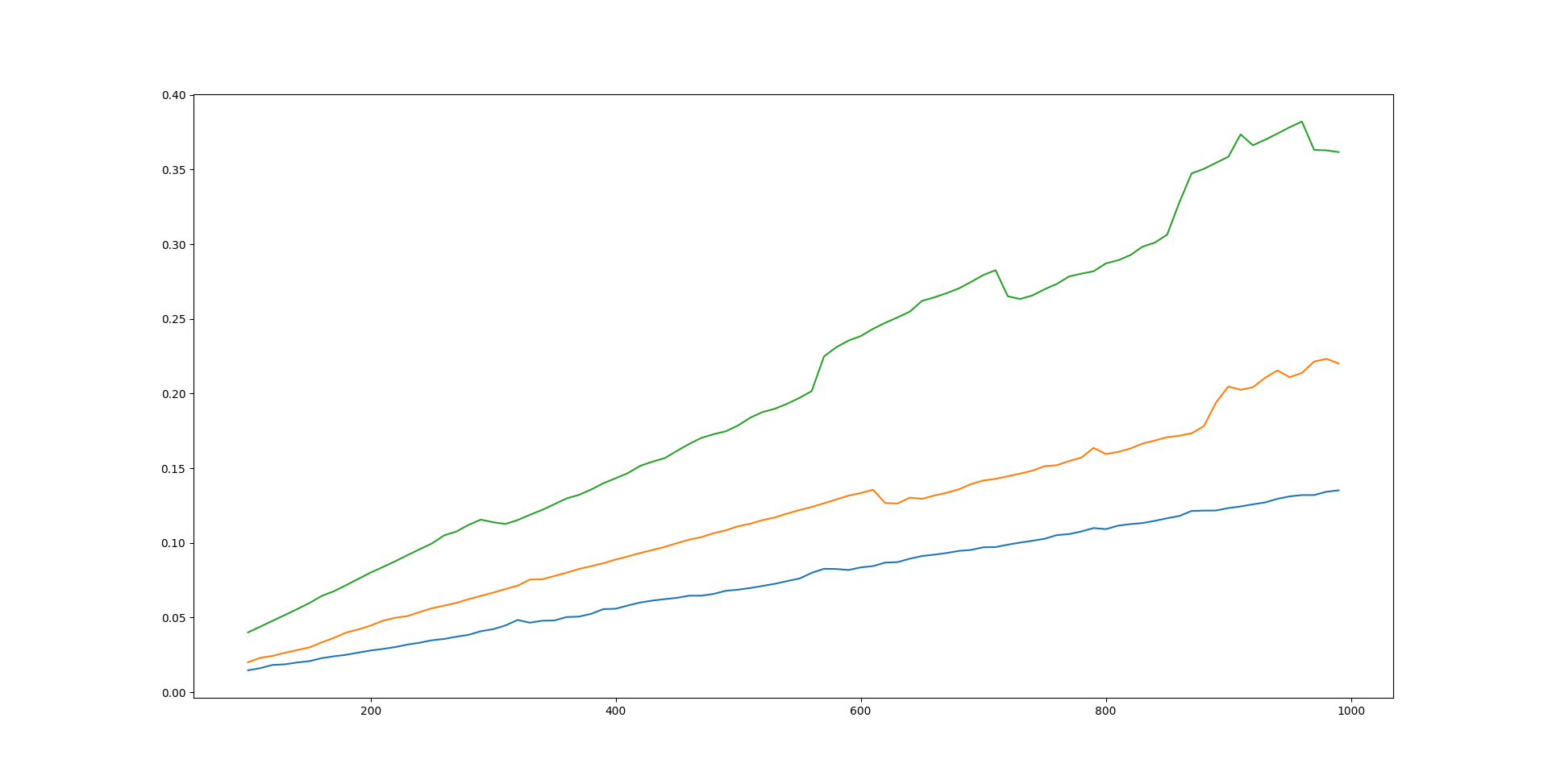

Supplement: Supplemental Information 2 — The simulation of the VANET environment. [file peerj-cs-07-714-s002.zip › VANet-Auth-master/plots/160vs256vs512.png]

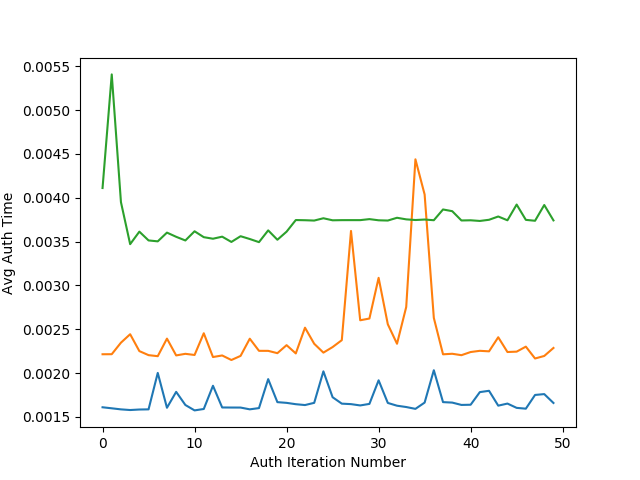

Supplement: Supplemental Information 2 — The simulation of the VANET environment. [file peerj-cs-07-714-s002.zip › VANet-Auth-master/plots/auth_160vs256vs512_sim_20_avg_50.png]
